# Barriers to translational research in Windsor Ontario: a survey of clinical care providers and health researchers

**DOI:** 10.1186/s12967-021-03097-6

**Published:** 2021-11-27

**Authors:** Justin B. Senecal, Karen Metcalfe, Kaila Wilson, Indryas Woldie, Lisa A. Porter

**Affiliations:** 1 Schulich School of Medicine and Dentistry, London, ON Canada; 2 WE-SPARK Health Institute, Windsor, ON Canada; 3 Cancer Program, Windsor Regional Hospital, Windsor, ON Canada; 4 Department of Biomedical Sciences, University of Windsor, Windsor, ON Canada

**Keywords:** Barriers, Translational research, Interdisciplinary collaboration, Research participation

## Abstract

**Background:**

Translational research is an ideology focussed on streamlining the transition of novel research into clinical practice to ultimately benefit populations. Central to this approach is overcoming barriers to research involvement and interdisciplinary collaboration. Identifying barriers has been the subject of several studies focused on communities with large academic hospitals. The Windsor-Essex region is currently built around community hospitals which have less of an emphasis on research, employ fewer physicians holding academic appointments and generally do not provide incentivised time for research and training. In this study, we surveyed clinicians and researchers working in Windsor-Essex to gain insight into barriers to translational research important to those working in smaller sized, community-based research networks.

**Methods:**

Using an anonymous close-ended Qualtrics survey distributed via email, we surveyed faculty members from The University of Windsor and clinical care providers from Windsor-Essex (n = 68). This included 24 physicians, 14 allied health professionals, and 30 non-clinician researchers.

**Results:**

Managing competing interests, lack of time, funding, infrastructure, and networks were identified by greater than 75% of participants as barriers to research involvement. 62% of physicians identified the lack of permanent post-graduate medical trainees as a barrier. Clinicians were consistently less experienced in research skills compared to others; particularly in publishing results and applying for funding (p < 0.001). Schedule incompatibility, funding issues and identifying interested collaborators with overlapping interests were identified as barriers to interdisciplinary collaboration by 80% of participants. Moreover, 46% of those surveyed were unhappy with their research involvement and these individuals were 13% more likely to perceive research as important for their career progression (p = 0.244).

**Conclusions:**

This study identifies several important barriers to translational research in Windsor-Essex and suggests that many motivated researchers are unhappy with their current involvement. These results will inform decision making in the research community of Windsor-Essex and provides insight for communities of similar size and research capacity. Ultimately, enabling the translation of clinical research in all communities is required to ensure equitable access to cutting edge care.

**Supplementary Information:**

The online version contains supplementary material available at 10.1186/s12967-021-03097-6.

## Background

Windsor, ON is the third-most populous city in Southwestern Ontario. It is home to the 7th largest community teaching hospital, Windsor Regional Hospital (WRH), a post-acute community hospital Hôtel-Dieu Grace Healthcare, and the 14th largest university by enrollment, The University of Windsor (UoW), in the province [1, 2]. The city also hosts the Schulich School of Medicine and Dentistry’s lone distributed medical campus and permanent postgraduate medical trainees in family medicine and psychiatry. The health research community continues to grow, with the new translational research institute WE-SPARK Health Institute recently launching in the spring of 2020. Still, compared to the largest research networks in the country, the health research capacity is limited.

Barriers to participation in health research and interdisciplinary collaboration have been the subject of many studies, often in the context of identifying barriers to translational research (TR) [3]. TR is often described as “bench to bedside” and is focussed on streamlining novel research findings into widespread clinical changes [4, 5]. Central to this ideology is a multidisciplinary approach, requiring the input of both clinical care providers and graduate trained researchers. These two groups often experience different barriers to their research goals; likely due to their different educational backgrounds and professional responsibilities [6].

The generalizability of previous studies are questionable for the following reasons: past studies often take place in large, academic centers [3, 7, 8], they often only include those heavily involved in research [3, 7, 9, 10], and the resulting barriers are broad and difficult to interpret [3]. Unlike academic centres, community centres like WRH have less research funding, employ many physicians that lack academic appointments and do not generally provide incentivised protected time for research or training; though a precise definition is lacking [11]. While most clinical and translational research conducted in Canada takes place in these large academic centres, the benefits of conducting research in community hospitals like WRH is substantial for both researchers and patients [11]. Given the limitations of previous work, we set out to examine which TR barriers are important to a smaller research community and community-based hospitals. We surveyed clinicians and health researchers from Windsor-Essex and specifically examined participants’ confidence in research tasks, opinions on TR, barriers to health research participation and interdisciplinary collaboration. We identified several major barriers to research and collaboration in our community and found that those struggling with their research involvement perceived barriers differently. Our findings will inform decision-making in the Windsor-Essex research community and contribute to the understanding of TR barriers in smaller centres.

## Methods

### Survey design

We designed an Internet-based survey using Qualtrics, an established survey provider, to examine research experience, opinions on TR, barriers to participation in health research and barriers to interdisciplinary collaboration in Windsor-Essex. The survey was anonymous and close-ended. We first asked participants about their professional backgrounds; with a focus on identifying those with clinical care responsibilities. We also asked participants about their satisfaction with research involvement, faculty appointments, time spent on research and research area. We then divided participants into the following groups: clinical care provider vs non-clinicians, and happy vs unhappy with current research involvement. Much of the questionnaire was adapted from previous studies that explored these barriers in other locations [7–9, 12]. Others were designed by the research team based upon their experiences working in Windsor-Essex. We choose not to collect certain demographics, such as age, gender, and specific research area, as these are beyond the scope of the study and could potentially allow for individual participants to be discerned in our relatively small research community.

We provided participants with a list of barriers to research participation and interdisciplinary collaboration identified in previous studies [8, 12] and asked them to rate the impact of each barrier with the following Likert Scale: Not a barrier (0), Moderate Barrier (1), Major Barrier (2). Clinical care providers were asked about barriers to collaborating with non-clinical care providers and vice versa. Participants that have experienced such collaboration in the past were also provided a list of benefits and selected which benefits they experienced. We also asked participants to rank their confidence in a variety of research tasks (adapted from [12]) using the following Likert Scale: No (1), Little (2), Some (3), Moderate (4) and Very (5) Experienced.

To assess participants opinions on research productivity and TR, we provided participants with a list of research metrics and achievements (adapted from [7, 9]) and asked them to choose no more than 4 that were relevant to their careers. We then provided them with a series of statements on TR and asked them to rank their agreement using this Likert Scale: Strongly disagree (1), Disagree (2), Neither agree or disagree (3), Agree (4), Strongly Agree (5)**.**

### Study recruitment

Between July 2nd 2020 and Nov. 30th 2020, participants were recruited via email and community newsletter. Standardized emails including the survey link were sent to faculty members from the Faculty of Arts and Humanities, Kinesiology, Engineering, Science and Nursing at the UoW by their respective deans. Clinical care providers working at Windsor Regional Hospital were distributed emails via the Research Office with permission from the Chief of Staff. The link was also included in newsletters at WRH, UoW and WE-SPARK.

Participants were included in the study if they satisfied the following criteria: (1) the participant worked in Windsor-Essex, (2) was a clinical care provider or had research interests that “May have implications for healthcare policy, clinical care, treatment development or clinical education”, and (3) completed greater than 2/3 of the survey.

### Statistical analysis

Data from the questionnaire was imported into Excel 2020 (Microsoft Corporation, USA). Descriptive statistics (Likert values, proportions, frequency counts) were used to capture demographic data for the study population; as well as perceptions on barrier to research and interdisciplinary collaboration, confidence in research skills and opinions on TR. Statistical comparisons between groups (clinical care providers vs non-clinicians; unhappy vs happy with research involvement) was performed with an independent, unpaired t-test. Results were considered significant if p < 0.05.

### Ethics statement

This study received clearance from the Research Ethics Board of the UoW and WRH (REB# 37036). Informed consent was obtained from participants before they began the survey.

## Results

### Study participants and response rate

To assess which barriers to health research participation and interdisciplinary collaboration were important to those working in Windsor-Essex, we recruited clinicians from the area and faculty members from UoW to participate in our survey. 88 respondents completed some of the survey. 20 did not meet the inclusion criteria, leaving 68 participants that were included in the study. 10 of the included participants submitted partial surveys that were greater than 66% complete, the rest were completed in entirety. Amongst faculty members from the targeted faculties at the UoW, we received 40 responses from an estimated 447 members [Response Rate (RR) = 9%]. 24 physicians from WRH responded out of an estimated 485 physicians (RR = 5%). 14 allied health professionals also contributed, but we are unable to estimate a total number of these professionals that were recruited. A total RR is likely higher than each individual RR combined due to physicians that are also UoW faculty.

As seen in Table 1, 38 (56%) of the participants were clinical care providers and 30 (44%) were not. Of the clinical care providers, 24 (63%) were physicians and 14 (37%) were allied health professionals, including nurses, social workers, and physiotherapists. 87% of clinical care providers spent less than 20% of their time on research compared to only 10% of non-clinicians. 90% of participants that were non-clinicians had a graduate degree, compared to only 43% of clinicians. Participants carried out a variety of research tasks and had a variety of research interests, but clinical research was the most common, particularly amongst clinical care providers (Additional file 1: Figure S1).

**Table 1:** Characteristics of survey respondents n = 68

	All	Clinical care provider	Non-clinician	Happy with research involvement	Unhappy with research involvement
Total	68 (100%)	38 (56%) Physician: 24 (35%) *AHP*: 14 (20%)	30 (44%)	31 (46%)	31 (46%)
< 20% time spent on research	36 (53%)	33 (87%)	3 (10%)	14 (45%)	17 (54%)
PhD	33 (48%)	6 (16%)	27 (90%)	18 (58%)	13 (42%)
No graduate degree	25 (37%)	22 (57%)	3 (10%)	6 (19%)	11 (35%)
MD	24 (34%)	24 (63%)	0 (0%)	10 (32%)	9 (29%)
University of Windsor	35 (52%)	11 (29%)	24 (80%)	17 (54%)	15 (48%)
Schulich School of Medicine and Dentistry	18 (26%)	17 (45%)	1 (3%)	6 (19%)	8 (26%)
No Faculty Affiliation	16 (23%)	11 (29%)	5 (17%)	8 (26%)	7 (22%)
St. Clair College	2 (3%)	2 (3%)	0 (0%)	1 (3%)	1 (3%)
Unhappy With Research Involvement	31 (46%)	20 (53%)	11 (37%)	N/A	N/A

### Research satisfaction and career development

To assess whether participants were satisfied we asked whether they agreed with the statement “I am happy with my current research involvement.” 46% of participants were unhappy with their current research involvement, including 53% of clinical care providers and 37% of non-clinical care providers (Table 1). We then asked participants whether they felt research was important for their career progression (Additional file 1: Figure S2). Interestingly, those that were unhappy with their research involvement were more likely to state that research was important for their career progression when compared to those that were happy with their current research (83% vs 70%, p = 0.244; Additional file 1: Figure S2).

### Barriers to research participation

**Fig. 1:**
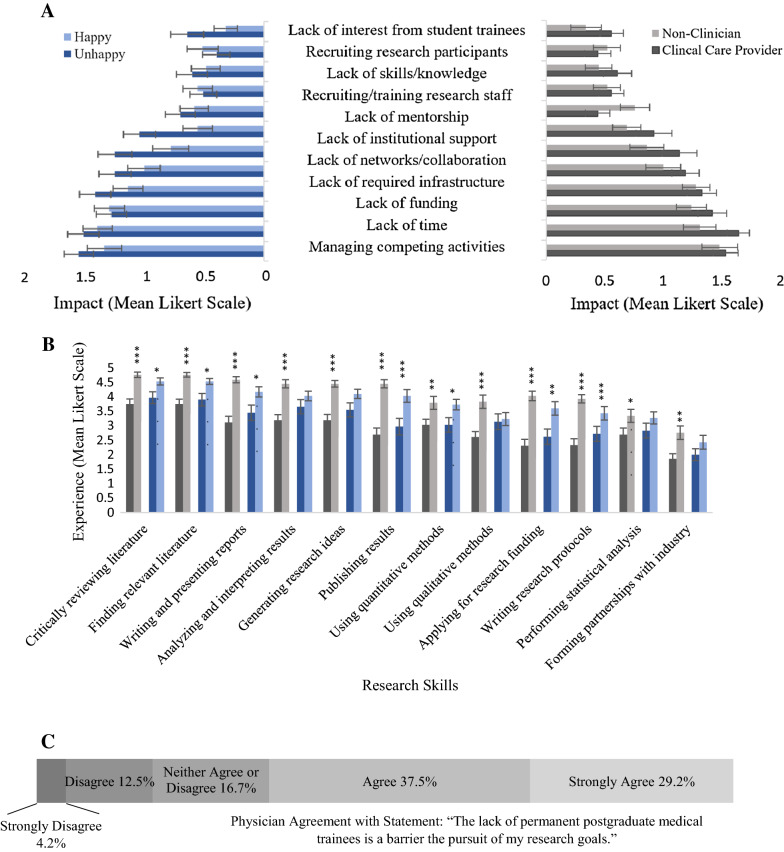
Barriers to research participation and experience in research skills. Participants were divided by clinical responsibility (clinical care provider vs non-clinician) and satisfaction with research involvement (happy vs unhappy). Significant differences between groups determined by unpaired t-test; error bars represent SE. **A** Impact of barriers to research participation rated by mean Likert scale: Not a barrier (0), Moderate barrier (1), Major barrier (2). **B** Self-perceived experience with various research tasks, rated by mean Likert scale: No (1), Little (2), Some (3), Moderate (4) and Very (5) Experienced. **C** Impact of absence of permanent postgraduate medical trainees on physician research goals; measured by proportion of physicians (n = 24) who strongly disagreed, disagreed, neither agreed or disagreed, agreed or strongly agreed with the above statement

To determine which barriers to research participation were important to those working in Windsor-Essex, respondents were asked to rank the impact of various barriers using a Likert Scale (Fig. 1A). Managing competing activities, lack of time, funding and infrastructure were the most impactful barriers in the opinion of the participants (Fig. 1A); with more than 85% of participants identifying each as a moderate or major barrier. Clinical care providers and non-clinicians perceived the impact of each barrier as relatively equal, with the largest discrepancy being that non-clinicians perceived recruiting and training research staff as significantly more impactful than clinicians (p = 0.0135; Fig. 1A). Those that were unhappy with their research involvement identified lack of institutional support and mentorship as significantly more impactful than those that were happy with their research involvement (p < 0.05, Fig. 1A).

We also asked participants how experienced they were in a variety of common research tasks using a Likert Scale (Fig. 1B). Clinicians perceived themselves as significantly less experienced than non-clinicians in all research tasks we included (p < 0.01, Fig. 1B). The largest differences were in publishing results, applying for research funding, and writing research protocols (Fig. 1B). There was a similar trend when participants were divided by their satisfaction with research involvement, with those that were happy generally feeling more experienced than those who were unhappy. However, the differences were not as large (Fig. 1B). 62% of physicians surveyed also felt that the lack permanent postgraduate trainees in the area was a barrier to their research goals (Fig. 1C).

### Barriers to interdisciplinary collaboration

**Fig. 2:**
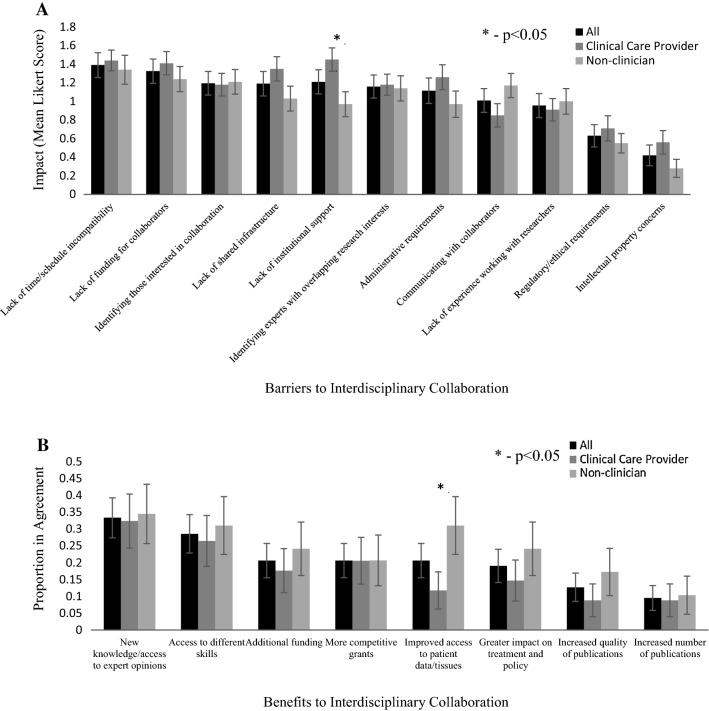
Benefits and barriers to interdisciplinary collaboration. Participants were divided by clinical responsibility (clinical care provider vs non-clinician). Significant differences between groups determined by unpaired t-test; error bars represent SE. **A** Impact of barriers to interdisciplinary collaboration rated by mean Likert scale: Not a barrier (0), Moderate barrier (1), Major barrier (2). **B** Proportion of participants (with experience in interdisciplinary collaboration) in agreement with each benefit

We asked clinicians to rank the impact of various barriers to collaboration with non-clinicians and vice versa using a Likert scale. Most barriers were equally impactful to clinical care providers and non-clinicians, with greater than 80% of participants identifying schedule incompatibility, lack of funding, identifying interested collaborators and lack of shared infrastructure as barriers (Fig. 2A). Clinicians felt that lack of institutional support was more impactful than non-clinicians, and it was the most impactful barrier identified by this group (p = 0.0115, Fig. 2A).

38% of clinical care providers in our study have collaborated with non-clinicians on research tasks and 40% of non-clinicians have collaborated with clinicians. Interestingly, all barriers to interdisciplinary were ranked as more impactful by those that had experienced interdisciplinary collaboration (NS; Data not shown). Participants who have experienced interdisciplinary collaboration were asked to identify what benefits they experienced (Fig. 2B). Access to expert opinions/new knowledge, different skills and additional funding were the most frequently cited benefits, while increased publications was the least frequently identified benefit (Fig. 2B). Non-clinicians were significantly more likely to cite improved access to patient data or tissues as a benefit when compared with clinicians (31% vs 12%; p = 0.049). This was the only significant difference between the two groups.

### Opinions on research productivity and TR

**Fig. 3:**
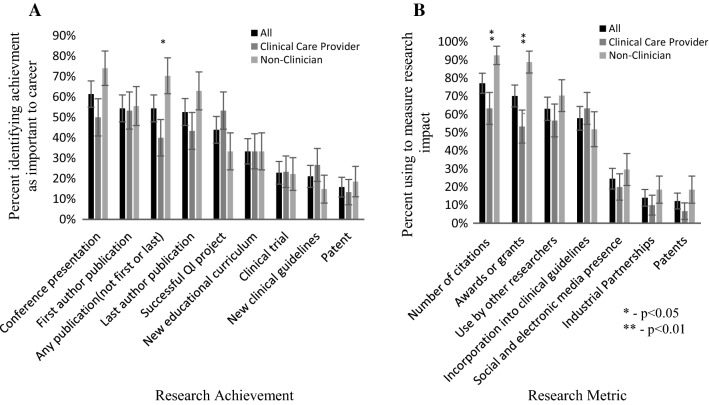
Opinions on Research Productivity. Participants were divided by clinical responsibility (clinical care provider vs non-clinician). Significant differences between groups determined by unpaired t-test; error bars represent SE. **A** Proportion of participants identifying research achievements that were important for their career. Participants were able to select no more than four from the list provided. **B** Proportion of participants using the listed research metrics to measure the impact of their research. Participants were able to select no more than four from the list provided

To assess the participants’ opinions on research productivity, we asked each to identify which metrics and achievements were important to them. Traditional achievements, including conference presentations and publications, were identified by more than 50% of our participants as important for career progression (Fig. 3A). Clinicians were more likely to identify first author publications as more important than other publications (Fig. 3A). Publications that were neither first nor last author were identified as important by non-clinicians more so than clinicians (70% vs 40%; p = 0.0189). Non-clinicians were significantly more likely to use number of citations and awards/grants to measure the impact of their research (p < 0.01; Fig. 3B).

**Fig. 4:**
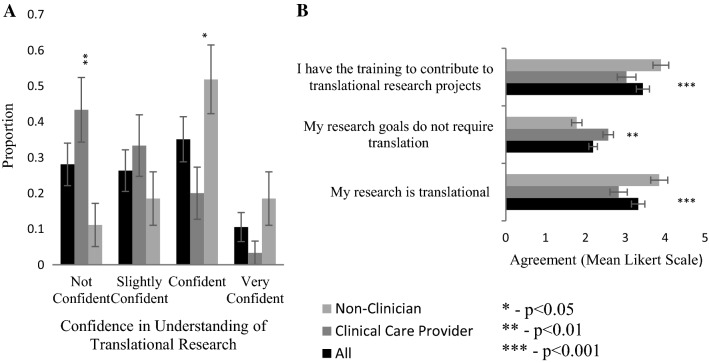
Opinions on Translational Research. Participants were divided by clinical responsibility (clinical care provider vs non-clinician). Significant differences between groups determined by unpaired t-test; error bars represent SE. **A** Participant confidence in understanding of translational research. **B** Participants were asked whether they agree with the listed the statements. Agreement of each group was measured via mean Likert scale: Strongly disagree (1), Disagree (2), Neither agree or disagree (3), Agree (4), Strongly Agree (5)

We next asked participants how confident they were in their understanding of TR. 46% of participants were either confident or very confident in their understanding of TR (Fig. 4A). Clinicians were more likely to lack confidence than non-clinicians (43% vs 11%; Fig. 4B). Using a Likert scale to rate agreement with statements about TR, we found that fewer clinical care providers felt that their research would be considered translational or had the training to participate in translational projects as compared to non clinicians (p < 0.001; Fig. 4B). Most of the participants were unsure whether their research required translation, however, clinicians were less likely to feel that their research goals required translation (p = 0.0029; Fig. 4B).

## Discussion

**Fig. 5:**
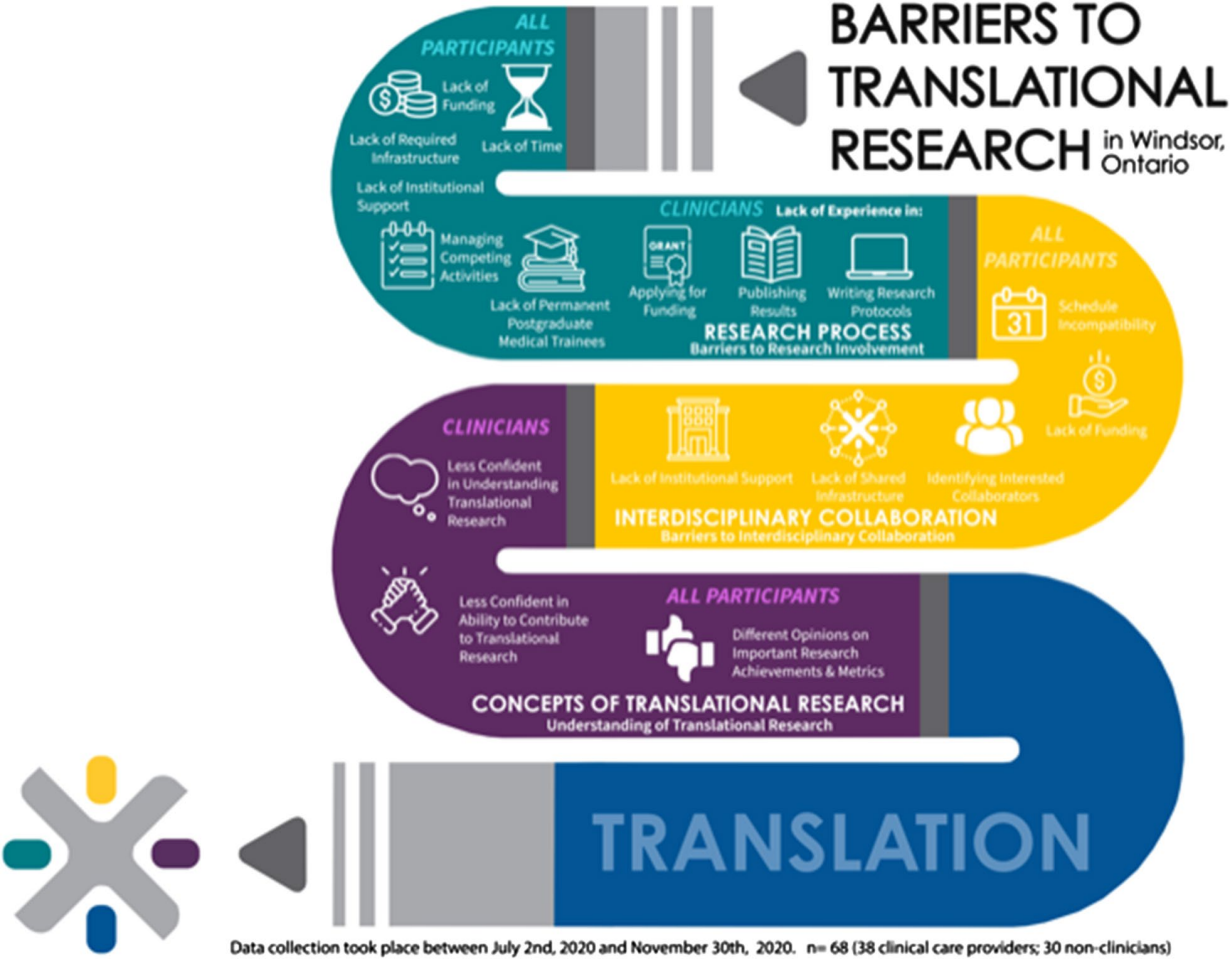
Barriers to Translational Research in Windsor, ON. Summary of barriers to translational research important to clinicians and non-clinicians surveyed in our study (n = 68). Barriers are sorted into three of the thematic barriers to translational research broadly identified initially by Fudge et al*.* [3]; including “Research Process”, “Interdisciplinary Collaboration” and “Concepts of Translational Research.”

In this study, we surveyed health researchers from a mid-sized comprehensive University that lacks a full medical school campus to assess the barriers to research participation and interdisciplinary collaboration. To our knowledge, this is the first such study in a smaller, Canadian research community that contains only community hospitals. Key findings are summarized in Fig. 5. We also sorted our findings into 3 of the 5 thematic barriers identified in the narrative synthesis by Fudge et al. [3]; including “Research Process”, “Interdisciplinary Collaboration” and “Concepts of Translational Research” (Fig. 5).

We included participants with various research interests from clinical and non-clinical backgrounds across a range of disciplines; reflecting the growing group of professionals that contribute to health research [4] (Additional file 1: Figure S1, Table 1). 46% of study participants felt unhappy with their current research involvement and 83% of these individuals felt that research was important for their career progression (Additional file 1: Figure S2)(Table 1). This suggests that there is a group of motivated but dissatisfied researchers in this community. This group felt that lack of mentorship and institutional support were significantly more impactful than those that were happy with their current research involvement (Fig. 1A). The data agrees with a survey of Canadian respiratory workers, which suggested that lack of mentorship was a more important barrier for those not involved in research when compared to those actively engaged in research [12]. Clinicians and non-clinicians ranked barriers to research participation relatively equally (Fig. 1A), with more than 85% of participants selecting managing competing activities, lack of time, funding, and infrastructure as a barrier. These barriers have been frequently cited as important to researchers in studies from other geographic areas [8, 10, 12, 13].

Study participants with clinical responsibilities were significantly less confident than non-clinicians in research skills, with the largest disparity being in applying for research funding and publishing results (Fig. 1B). Previous studies have also suggested that this lack of confidence could be a barrier to participating in interdisciplinary collaboration, particularly with colleagues who are more research focussed [9, 12]. Clinicians and non-clinicians generally agreed on the benefits and barriers to interdisciplinary collaboration (Fig. 2A); with 80% identifying schedule incompatibility, lack of funding, identifying interested collaborators and lack of shared infrastructure as a moderate or major barrier. Both groups also tended to find traditional research metrics, such as publications, presentations, and citations, as the most significant metric of research productivity (Fig. 3). This is in contrast to previous work which suggested that clinical researchers found incorporation into clinical guidelines as more important than publications [14]. Furthermore, non-clinicians were more confident in their understanding of TR and largely felt that they had the necessary training to contribute to TR. Non-clinicians also felt that their research was either already translational or requires translation (Fig. 4).

A strength of this study is our broad sample that is inclusive of participants with different research commitments, backgrounds, and clinical responsibilities. However, this sampling strategy did lead to a relatively low response rate and potential response bias. Physicians in particular are known to be difficult to survey, with previous studies reporting response rates as low as 2.7–11.4% [15, 16]. In addition, our strategy likely selects for participants with an interest in translational research, which may be a smaller proportion of individuals in a community-based research environment. By dividing participants based on their satisfaction with their research involvement, we assured that our findings weren’t biased by those with particularly favourable or unfavourable views of the research community.

While the close-ended nature of the survey makes drawing conclusions difficult, our data suggests that mentorship and assistance with obtaining grants would benefit researchers in Windsor-Essex. Plans to develop a database of interested researchers to aid with identification of interested collaborators are already underway in our community. Other barriers, such as managing competing activities and schedule incompatibility, are more difficult to address as they are ingrained in the culture of various careers. Future studies should allow for narrative responses to better identify problems and potential solutions.

## Conclusion

In summary, we found that while clinicians and non-clinicians from Windsor-Essex perceive similar barriers to research participation and interdisciplinary collaboration, they differ in terms of their confidence in research skills and their opinions on TR. Lack of mentorship, and institutional support were more important barriers to those that were dissatisfied with their current research involvement; but future study is needed to better define these barriers. These findings will inform decision making in Windsor-Essex and similarly sized research communities that are often neglected in these studies.

## Supplementary Information

**Additional file 1.** Supplemental Figures and the Distributed Questionnaire.

## Data Availability

All data generated or analysed during this study are included in this published article [and its additional information files].

## Abbreviations

AHP: Allied Health Professional
RR: Response rate
TR: Translational research
UoW: University of Windsor
WRH: Windsor Regional Hospital
