# Supplementary material for: Barriers to translational research in Windsor Ontario: a survey of clinical care providers and health researchers
Source: J Transl Med. 2021 Nov 27;19:479. doi: 10.1186/s12967-021-03097-6 (PMC8626997; doi:10.1186/s12967-021-03097-6)
Supplement: Supplementary file 1 — Additional file 1. Supplemental Figures and the Distributed Questionnaire. [file 12967_2021_3097_MOESM1_ESM.docx]

**Additional Information/Figures:**

**Figure S1. Research interests of study participants.**

1. Proportion of participants with research interests in each of the listed categories. Participants selected as many as applicable; error bars represent SE. No statistical analysis.
2. Proportion of participants who carry out each of the listed research tasks. Participants selected as many as applicable; error bars represent SE. No statistical analysis.

**B**

**A**

p=0.244

p=0.067

**Figure S2. Importance of Research Productivity for Career Progression.** The proportion of individuals that agreed with the statement “research productivity is important for my career progression.” Participants were divided by clinical responsibility (clinical care provider vs non-clinician) and satisfaction with research involvement (happy vs unhappy). Statistical analysis by unpaired t-test; error bars represent SE.

**Questionnaire:**

Q1. If you understand the information provided and wish to proceed voluntarily with the survey, please indicate so below. Partial responses will be recorded. Upon completion of the survey, you will be given the option to submit your responses or have them deleted.

- I consent to the voluntary participation of this study.
- I do not consent to the voluntary participation of this study.

Q2 Have you previously completed this survey?

- Yes
- No

Q3 Do you currently work in the Windsor-Essex area?

- Yes
- No

Q4 Do you currently work as a physician?

- Yes
- No

Q5 Do you currently work as a clinical care provider? 
In order to protect your confidentiality, please do not include any identifying information in any of the following questions.

- Yes (Please Specify) ________________________________________________
- No

Q6 Are you a faculty member at any of the following institutions? (Select all that apply)

- University of Windsor
- St. Clair College
- Other
- I am not a faculty member at any institution

Q7 Please indicate which of the following degrees you possess. Select all that apply.

- Bachelor's
- Master's
- PhD
- MD
- Other (Please Specify) ________________________________________________

Q8 Do you participate in research or any research related tasks?

- Yes
- No

Q9 What percentage of your time would you estimate you spend on research/research related tasks?

- <20%
- 21%-40%
- 41%-60%
- 61%-80%
- >81%

Q10 Does your research have any potential implications for healthcare policy, clinical care, treatment development or clinical education?

- Yes
- Maybe
- No

Q11 What type(s) of research are you currently involved with? (Select all that apply).
In order to protect your confidentiality, please do not include any identifying information in any of your responses.

- Clinical
- Epidemiology
- Basic Science
- Social Science
- Engineering
- Statistics
- Translational
- Education
- Healthcare policy/Public Health
- Other (Please Specify) ________________________________________________

Q12 Please select any of the following research related tasks you have performed or been involved with (if applicable). Select all that apply.

- Collecting patient tissues
- Analyzing patient tissues
- Collecting patient data
- Analyzing patient data
- Drug Screening
- Animal research
- Clinical Trials
- Studying educational curriculum
- Implementing new education programs/curriculum
- Implementing new treatments/procedures, treatment guidelines, screening tools or health programs
- Other (Please Specify) ________________________________________________

Q13 Please identify which of the following have been a barrier to the pursuit of your research goals.

|  | Not a Barrier (0) | Moderate Barrier (1) | Major Barrier (2) |
| --- | --- | --- | --- |
| Lack of mentorship |  |  |  |
| Lack of funding |  |  |  |
| Lack of time |  |  |  |
| Lack of skills and knowledge |  |  |  |
| Lack of networks and collaboration |  |  |  |
| Lack of interest from student trainees |  |  |  |
| Lack of institutional support |  |  |  |
| Lack of required infrastructure |  |  |  |
| Regulatory/Ethics Approval |  |  |  |
| Recruiting Research Participants |  |  |  |
| Recruiting/training research staff |  |  |  |
| Managing competing activities (clinical, administrative, teaching, etc.) |  |  |  |

Q14 Please state how much you agree with the following statements:

|  | Strongly agree | Somewhat agree | Neither agree nor disagree | Somewhat disagree | Strongly disagree |
| --- | --- | --- | --- | --- | --- |
| I am happy with my current amount of research involvement |  |  |  |  |  |
| Research productivity is important to my career progression |  |  |  |  |  |

Q15 The lack of permanent postgraduate medical trainees (residents) is a barrier to the pursuit of my research goals. (Asked only of physicians)

- Strongly agree
- Somewhat agree
- Neither agree nor disagree
- Somewhat disagree
- Strongly disagree

Q16 Please state how much experience you have in the following research skills.

|  | No Experience | Little Experience | Some Experience | Moderate Experience | Very Experienced |
| --- | --- | --- | --- | --- | --- |
| Writing research protocols |  |  |  |  |  |
| Applying for research funding |  |  |  |  |  |
| Generating research ideas |  |  |  |  |  |
| Finding relevant literature |  |  |  |  |  |
| Critically reviewing literature |  |  |  |  |  |
| Using quantitative methods |  |  |  |  |  |
| Using qualitative methods |  |  |  |  |  |
| Performing statistical analysis |  |  |  |  |  |
| Analyzing and interpreting results |  |  |  |  |  |
| Writing and presenting reports |  |  |  |  |  |
| Publishing results |  |  |  |  |  |
| Forming partnerships with industry |  |  |  |  |  |

Q17 Have you collaborated with non-clinician professionals in Windsor on research tasks?

- Yes
- No

Q18 Have you collaborated with clinicians in Windsor on research tasks previously?

- Yes
- No

Q19 What were the benefits of this type of collaboration? Select all that apply. 
In order to protect your confidentiality, please do not include any identifying information in any of your responses.

- Additional funding
- More competitive grants
- Increased quality of publications
- Increased number of publications
- Greater impact on treatment and policy
- Improved access to patient data/tissues
- New knowledge/access to expert opinions
- Access to different skills

Q20 Please identify which of the following have been a barrier to collaborating with (non-clinician/clinician) researchers in Windsor.

|  | Not a Barrier | Moderate Barrier | Major Barrier |
| --- | --- | --- | --- |
| Identifying experts interested in collaboration |  |  |  |
| Identifying experts with overlapping research interests |  |  |  |
| Communicating with collaborators |  |  |  |
| Lack of time/schedule incompatibility |  |  |  |
| Lack of funding for collaborators |  |  |  |
| Lack of experience working with (non-clinician/clinician) |  |  |  |
| Administrative requirements |  |  |  |
| Regulatory/ethical requirements |  |  |  |
| Lack of shared infrastructure |  |  |  |
| Lack of institutional support |  |  |  |
| Intellectual property concerns |  |  |  |

Q21 Please select the 4 achievements that are most important for your career progression. If less than 4 are applicable, you may select less.

- First author publication
- Last author publication
- Any publication that is neither first or last author
- Conference Presentation
- Clinical trial
- Patent
- New clinical guidelines
- Successful quality improvement project
- New educational curriculum

Q22 Please select the 4 measures that you use most frequently to measure the impact of your research. If less than 4 are applicable, you may select less.

- Incorporation into clinical guidelines
- Number of researchers using my research
- Number of citations of previous publications
- Awards or grants
- Social and electronic media presence
- Patents
- Industrial Partnerships/Business Models

Q23 How confident are you in your understanding of translational research?

- Very Confident
- Confident
- Slightly Confident
- Not Confident

Q24 Please indicate how much you agree with the following statements:

|  | Strongly agree | Somewhat agree | Neither agree nor disagree | Somewhat disagree | Strongly disagree |
| --- | --- | --- | --- | --- | --- |
| My research is translational |  |  |  |  |  |
| My research goals do not require translation |  |  |  |  |  |
| I have the training to contribute to translational research projects |  |  |  |  |  |

Q25 Thank you for taking the time to complete this survey. Please indicate whether you would like to submit your survey responses or have them deleted.

- Submit my survey responses
- Do not submit my survey responses and delete my response
